# The heat of the battle: inflammation’s role in prostate cancer development and inflammation-targeted therapies

**DOI:** 10.1007/s12672-025-01829-4

**Published:** 2025-02-01

**Authors:** Ujjawal Sharma, Anidrisha Sahu, Himanshu Shekhar, Bunty Sharma, Shafiul Haque, Damandeep Kaur, Hardeep Singh Tuli, Astha Mishra, Faraz Ahmad

**Affiliations:** 1 Department of Human Genetics and Molecular Medicine, Central University of Punjab, Bhatinda, 151001 India; 2 Department of Biotechnology, Graphic Era (Deemed to be University), Dehradun, Uttarakhand India; 3 Research and Scientific Studies Unit, College of Nursing and Health Sciences, Jazan University, 45142 Jazan, Saudi Arabia; 4 School of Medicine, Universidad Espiritu Santo, Samborondon, 091952 Ecuador; 5 University Centre for Research & Development, University Institute of Pharmaceutical Sciences, Chandigarh University, Gharuan, Mohali, Punjab 140413 India; 6 Department of Bio-Sciences and Technology, Maharishi Markandeshwar Engineering College, Maharishi Markandeshwar (Deemed to be University), Mullana, Ambala, 133207 India; 7 Department of Optometry, Chitkara School of Health Sciences, Chitkara University, Rajpura, Punjab India; 8 Department of Biotechnology, School of Bio Sciences and Technology (SBST), Vellore Institute of Technology, Vellore, 632014 India

**Keywords:** Prostate cancer, Inflammation, NF-κβ, P13K/AKT, JAK/STAT, Inflammatory microenvironment

## Abstract

In prostate cancer (PC), chronic inflammation silently guides disease progression, playing a significant role. As a major global health concern, PC contributes to high mortality rates and rising new cases worldwide, highlighting the urgent need for research into the molecular mechanisms behind this disease. Notably, the persistence of inflammation actively promotes cancer development, including in PC. This review explores the complex relationship between inflammation and PC, examining the molecular pathways, genetic and environmental factors, and clinical implications involved in inflammation-driven carcinogenesis. From cellular and molecular elements of the inflammatory microenvironment to mechanisms like epithelial-to-mesenchymal transition (EMT), reactive oxygen species (ROS) generation, and inflammasome activation, these processes highlight inflammation’s influence on PC progression and metastasis. Furthermore, this review discusses current therapeutic strategies targeting inflammation in PC management and identifies future research directions aimed at unraveling the complexities of inflammation-induced PC (Supplementary Fig. 1). It defines the complex relationship between inflammation and PC, emphasizes the importance of targeting inflammation therapeutically and highlights innovative approaches in PC treatment.

**Graphical Abstract:**

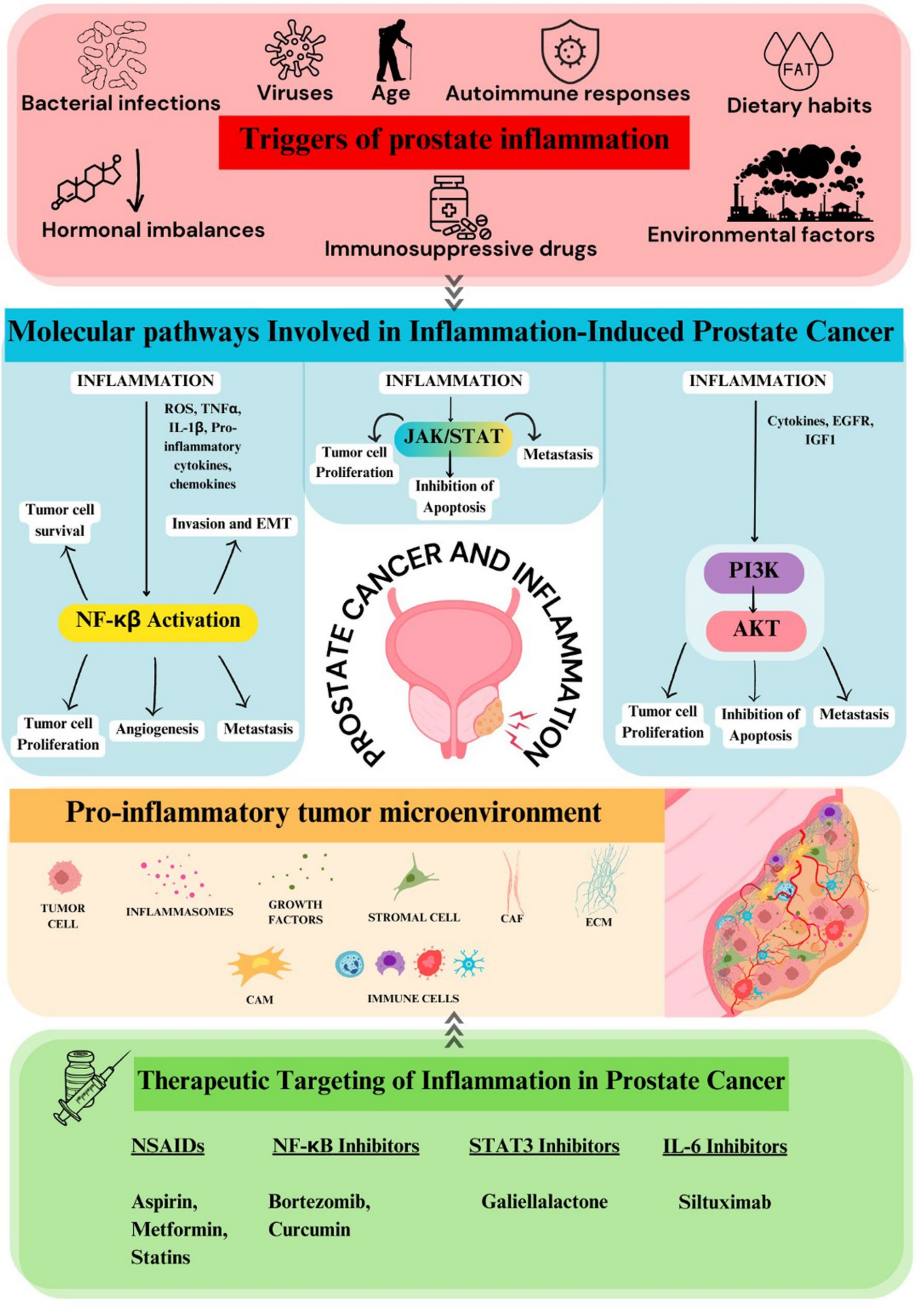

**Supplementary Information:**

The online version contains supplementary material available at 10.1007/s12672-025-01829-4.

## Introduction

The prostate is a small, apple-shaped gland present beneath the bladder in male. It serves as a trigger for ejaculation, helps sperm activation, capacitation and produces fluid that protects and nourishes sperm. Therefore, it plays significant role in male fertility. Additionally, the prostate functions as an immune-competent organ, comprising lymphocytes, macrophages, and mast cells, which helps fight infection in the prostate. These immune cells increase in number with age, which might make the prostate more prone to inflammation. While inflammation is a fundamental process essential for tissue repair, chronic inflammation in the prostate has been extensively linked to an increased risk of PC development and progression. Chronic prostatic inflammation is triggered by chemicals such as uric acid, released from the damaged tissue, which activates the immune cells for production of inflammatory cytokines that can cause the recruitment of additional inflammatory cells. Various factors, including bacterial or viral infections, dietary habits, hormonal imbalances, autoimmune responses, age, or certain medications, can contribute to chronic inflammation in the prostate. This persistent inflammation can lead to genetic and epigenetic changes that promote the development and progression of PC. Moreover, inflammatory cells release sequestered growth factors and recruit proangiogenic leukocyte subsets, which can promote angiogenesis, thereby supplying nutrients for the growth of tumor. This sustained inflammatory state creates a pro-tumorigenic microenvironment, promoting tumorigenesis.

PC is very frequently diagnosed in men aged 65–74 and ranks as the second most diagnosed cancer in men. It is also the 5th leading reason of cancer-related deaths among males, only after lung cancer. In the year 2020, about 1.4 million new PC incidents were reported, resulting in approximately 0.37 million deaths [1]. PC has a lifetime incidence of approximately 1 in 9 men, with a mortality rate of about 1 in 41 men, according to the National Cancer Institute. According to The Lancet Commission on PC, the incidence of new PC cases is expected to increase from 1.4 million in 2020 to 2.9 million by 2040; that is, the cases are going to double by 2040 [2]. These statistics emphasize the urgent need to improve our understanding of the disease and address the rising burden of this disease in the coming years.

## Pathophysiology of prostate cancer

The prostate is a small, apple-shaped gland, surrounding the urethra and is located just underneath the bladder. It serves as a switch between urine and ejaculation, aids in sperm activation and capacitation, and generates sperm-protecting and sperm-nourishing fluid. The prostate is primarily dependent on testosterone and its more powerful metabolite dihydrotestosterone (DHT), for proper functions. Furthermore, the prostate naturally contains inflammatory cells, and the number of these cells in the extracellular matrix and prostate tissue tends to rise with age, which may enhance the prostate's susceptibility to chronic inflammation [3]. These immune cells release cytokines, which cause a constantly active stroma, resulting in tumorigenesis. Furthermore, inflammatory cells attract proangiogenic leukocyte subsets and produce growth factors, which encourage angiogenesis and support tumor development.

The multifaceted nature of PC arises from the complex interplay of various molecular pathways that govern its initiation and progression. The androgen receptor (AR) signaling pathway is central to PC biology, increased expression and mutations of AR leads to acquiring agonistic properties of anti-androgens. epigenetic silencing of AR expression by DNA methylation, and ubiquitination degradation of AR by the proteasome, further causes disease progression [4]. PC is associated with the NF-κβ (p65) and Sonic Hedgehog pathways, these pathway’s activation leads to enhance cell survival, proliferation, and metastasis [5]. Through the activation of NF-κβ signaling, TNF-α (tumor necrosis factor-α) and IL-17 (interleukin-17) raise the expression of PD-L1 (programmed cell death protein 1) in LNCaP cells, hence promoting tumor cell motility and chemoresistance. Angiogenesis, tumor growth, and immune evasion are all impacted by the tumor microenvironment (TME) in PC [6]. The development of PC can be influenced by the stromal, immune, and endothelial cells found in the TME. Genetic variations associated with a higher risk of PC include SNPs in the IL1R2, IL8RB, and TLR4 genes. Five to ten percent of PC cases are caused by genetic mutations in genes involved in DNA mismatch repair, such as RNASEL, BRCA1, BRCA2, HOXB13, MSH2, MLH1, and others [7–9]. Epigenetic changes, including DNA methylation, histone methylation, acetylation, and others, as well as dysregulation of non-coding RNA, also have an impact on PC development. Tumor-stroma interactions and extracellular matrix (ECM) remodeling are also significant contributors to PC development. Understanding the complex relationships between inflammation and PC is essential to creating novel treatment strategies.

## Inflammation and prostate cancer

Inflammation is a fundamental element of our immune response to any kind of stress, such as injury, infection, etc. A growing number of studies have broadly examined the link between inflammation and cancer, supporting its role in promoting tumor initiation, progression, and metastasis. While acute inflammation has a protective role in eliminating harmful stimuli and initiating tissue repair, chronic inflammation creates a pro-tumorigenic microenvironment characterized by the sustained production of inflammatory mediators, including reactive oxygen species (ROS), chemokines, and cytokines. These molecules enhance the invasiveness and metastatic potential of cancer cells, stimulate cell proliferation, promote angiogenesis, and block apoptosis [10].

**Fig. 1:**
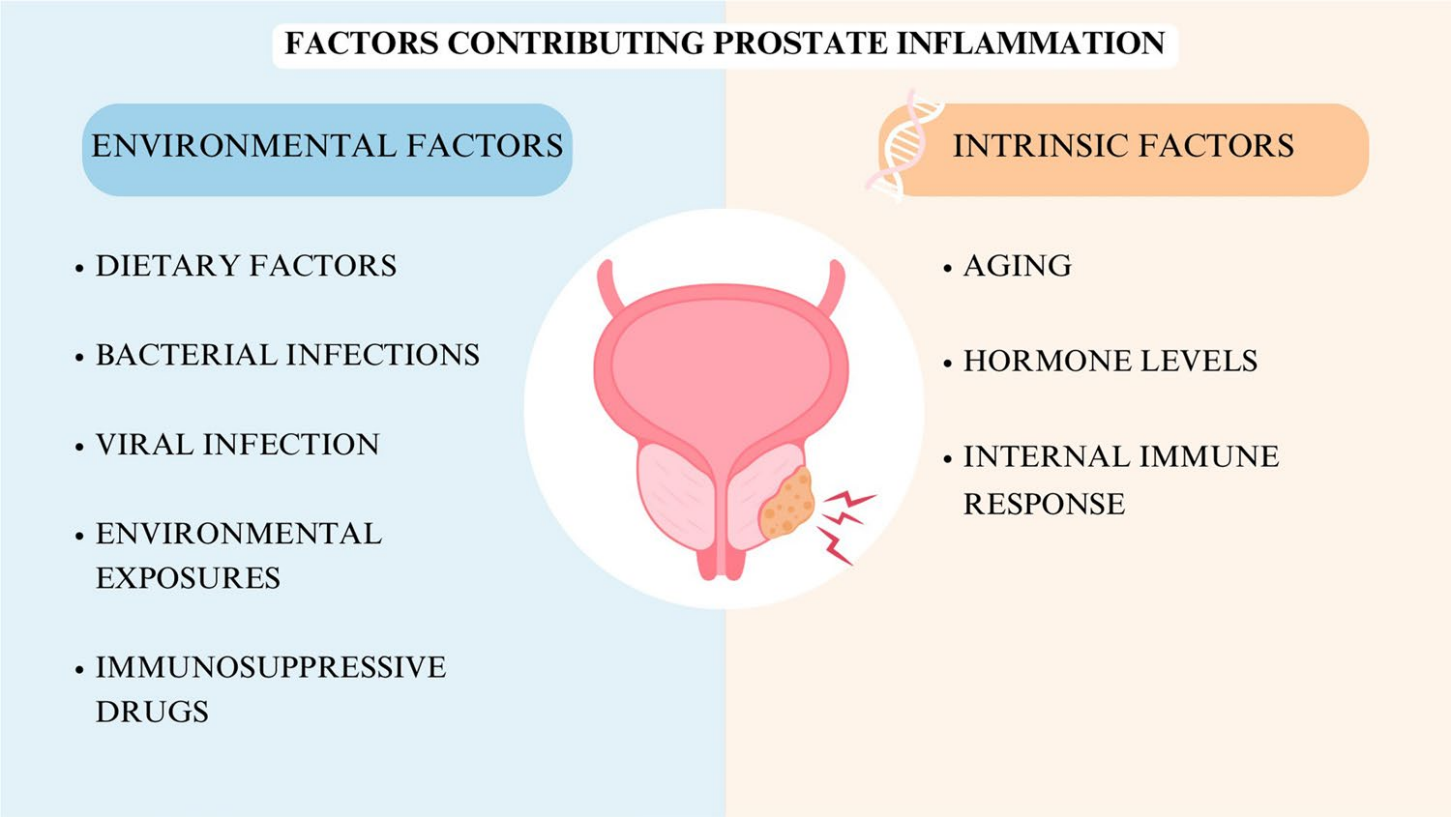
Factors contributing prostate inflammation: Various intrinsic and environmental factors contribute to the risk of chronic prostatic inflammation, including bacterial infections, viral infection, dietary factors, aging, hormonal imbalance, autoimmune responses, environmental exposers or certain medications

Chronic bacterial infections are a major factor that leads to chronic prostatitis, creating an inflammatory environment in the prostate, which can trigger cancer development [11]. Several intrinsic and environmental factors, such as bacterial infections, viruses, diet, age, hormones, autoimmune reactions, environmental exposure, and certain medications, increase the risk of chronic prostate inflammation (Fig. 1). Prolong inflammation caused by these factors can drive molecular changes that activate oncogenes or deactivate tumor suppressor genes, leading to the development and progression of PC.

### Factors contributing to chronic inflammation and the development of prostate cancer

Chronic bacterial infections are a major factor that leads to chronic prostatitis, creating an inflammatory environment in the prostate, which can trigger cancer development [11]. Several factors, such as bacterial infections, viruses, diet, age, hormones, autoimmune reactions, environmental exposure, and certain medications, increase the risk of chronic prostate inflammation (as seen in Fig. 1). These factors persist over time, causing genetic and epigenetic changes. These changes activate oncogenes (cancer-causing genes) and deactivate tumor suppressor genes, which drive PC development and progression. In addition, chronic inflammation can promote tumor heterogeneity through enhancing genetic divergence of tumor cells, such as TMPRSS2: ERG fusion. Inflammation has been proposed as a possible mechanism of this fusion by generating oxidative stress, which leads to damage in the form of double-strand breaks in DNA, ultimately enabling the occurrence of gene fusions [12]. Further inflammatory TME also serves as a selective pressure for tumor cell variants through genomic instability and heterogeneity [13]. As a result, there can be different subtypes of tumors that differ in aggressiveness, response to therapy, and tendency to metastasize.

#### Pathogenic infections

Bacterial infection contributes directly to chronic inflammation in the prostate, increasing the chances of PC. Common bacteria, including E. coli (55.5% of cases), Enterobacter cloacae, Klebsiella pneumoniae, Pseudomonas, and Aeruginosa, infect the prostate by entering through the urethra, infected urine backflow, or spreading from the rectum. These chronic bacterial infections can cause prolonged inflammatory cytokine production by chronic activation of NF- κβ, leading to tumorigenesis [14]. In a study by Sfanos in 2008, found that most prostatectomy samples (87%) contain bacterial DNA from one or more species. However, the majority of individual tissue core samples are negative, suggesting regional heterogeneity in the presence of bacteria. The presence of bacteria and resultant inflammation may create a diverse TME that drives the evolution of tumor subtypes [15].

Viruses also play a role in causing chronic prostate inflammation and increasing PC risk. HPV (Human Papillomavirus), EBV (Epstein-Barr Virus), HSV (Herpes Simplex Virus), XMRV (Xenotropic Murine Leukemia Virus-Related Virus), and polyomaviruses (BKV, JCV, and SV40) are among the viruses detected in prostate tissue with known cancer-causing potential [16].

#### Hormonal imbalances

Androgens, such as testosterone and dihydrotestosterone (DHT**)**, are known for playing a key role in normal growth and functioning of the prostate gland. These androgens are bound to androgen receptors (AR) in prostate cells, which regulates the cell growth and survival. And change in androgen levels is associated with initiation and progression of PC [17]. In PC, tumor growth is sustained through AR signaling activated by androgens. As a result, androgen deprivation therapy (ADT) is clinically used for PC treatment, with its mechanism involving the lowering of androgen levels or the blocking of AR activity. However, PC cells tend to preserve AR signaling even when androgen levels drop. They achieve this by upregulating AR expression, or producing AR variants that function without androgens [18]. These adaptations allow cancer to keep growing despite ADT, leading to castration-resistant PC (CRPC). Understanding these adaptive mechanisms is crucial for the development of more effective therapeutic strategies that can target the unique characteristics of each tumor.

#### Dietary habits

Consuming diets that are high in saturated fats, processed meats, have been reported significantly for increasing systemic inflammation, while consuming foods rich in omega-3 fatty acids have been seen to lower the risk of PC [19]. Obesity is associated with chronic low-grade inflammation and hormonal changes and this can increase risk for PC development [20]. In a study of 525 men recently diagnosed with PC, researchers found a connection between drinking high-fat milk and the progression of the cancer [21]. Moreover, a observational study found that intense exercise was linked to a decreased risk of advanced PC, as well as it decreases the chance of TMPRSS2 fusion-positive cases [22]. A gene set enrichment analysis of prostate tissue adjacent to tumors from same study indicated that immune pathways in the TME were altered in men who engaged in vigorous exercise compared to those who did not [23]. Recent research has shown that consuming a lot of macronutrients might raise oxidative stress and hence cause inflammation. It is important to talk about dietary carbohydrates because they can cause long-term effects from nutritionally driven oxidative stress [24]. Researchers have focused extensively on carbohydrate consumption because of its link to PC with high glycemic load or glycemic index diets [25]. A meta-analysis and systematic review and of 59 studies with 280,199 patients revealed that obesity raises the risk of PC-specific death by 19% and overall mortality by 9% [26]. Hyperinsulinemia is linked to various cancers, including PC. Studies, both in vivo and in vitro, have shown that high grade prostate tumors exhibit elevated levels of the insulin receptor-A isoform and a greater number of insulin receptors [27, 28]. These studies highlight the importance of diet, exercise, and metabolic health in preventing risk of PC.

#### Aging

Aging reduces tissue mass and decreases the functionality of adult stem cells in many tissues. However, in the case of prostate, the gland grows during two main periods: the first during puberty and the second in a man’s thirties. With the increase in age in men, the prostate typically enlarges, often reaching the size of a lemon by the age of 60. The exact cause for the prostate enlargement is unclear. Some researchers link this growth to an increase in luminal progenitor cells in the prostate.

#### Environmental exposures

Exposure to environmental toxins, pollutants, and carcinogens has been linked to prostatic inflammation and an increased risk of PC. Mainly, tobacco smoke and pesticides, contribute to this risk by generating excessive oxidative stress, leading to DNA damage, and PC development and malignancy [29]. Moreover, exposure to bisphenol A, benzo(a)pyrene, and ethyl-paraben have been associated with increased risk of PC [30].

#### Immunosuppressive medications

Immunosuppressive medications, including anti-inflammatory drugs (AIMs) like non-steroidal anti-inflammatory drugs (NSAIDs), immunosuppressants, and glucocorticoids, play a role in the development of PC, particularly in cases of metastatic PC. Some studies link NSAIDs, especially aspirin, to a lower risk of PC, while others suggest that using AIMs—particularly non-aspirin NSAIDs, Coxibs, and acetaminophen—may increase PC risk [31].

Therefore, identifying key regulators and pathways of inflammation is essential to understanding its role in PC and advancing treatment strategies.

### Molecular pathways involved in inflammation-induced prostate cancer

**Fig. 2:**
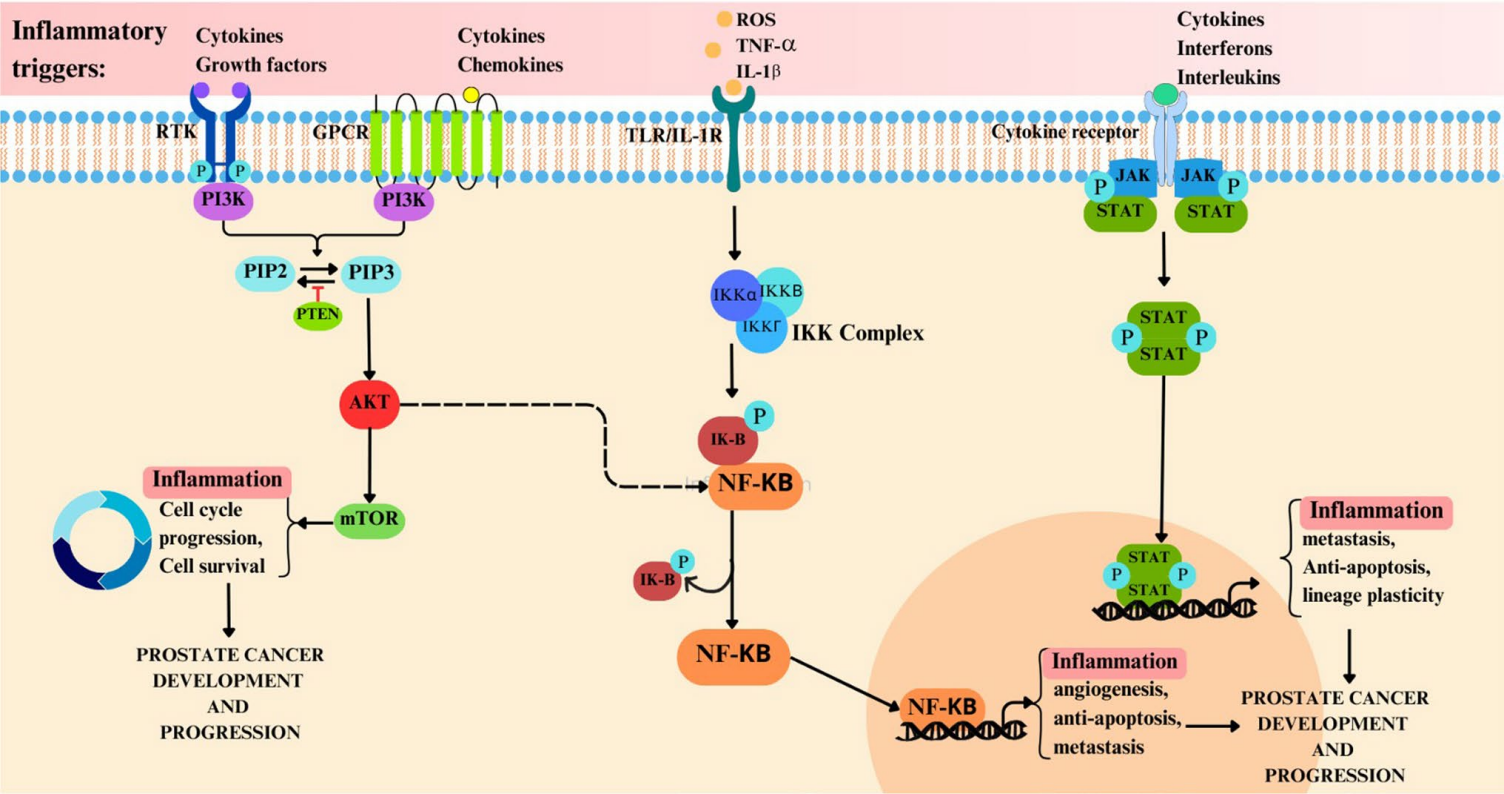
Inflammatory pathways implicated in prostate cancer: NF-κB, activated by inflammatory triggers like TNF-α, ROS among others, plays a central role in the release of cytokines, leading to chronic prostate inflammation that can lead to PC development. Similarly, the PI3K/AKT pathway, when activated, promotes the secretion of pro-inflammatory cytokines, contributing to the inflammation mediated PC. The JAK/STAT pathway, stimulated by cytokines, interferons, further drives inflammation, metastasis, and lineage plasticity, all of which are pivotal in the progression of prostate cancer

The development of cancer via chronic inflammation involves several pathways, including NF-κB, PI3K/AKT, and JAK/STAT. These pathways regulate immune responses, cell proliferation, survival, and metastasis. When these pathways become dysregulated, they can contribute to cancer progression (Fig. 2).

#### NF- κβ: a central player in inflammation and cancer

Both innate and adaptive immunity depend greatlyon NF-κβ, a major regulator of inflammatory reactions. NF-κβ controls the production of chemokines and cytokines, anti-apoptotic proteins, and adhesion molecules in immune cells and it is the key activator of many pro-inflammatory genes. Inflammatory triggers that activate this pathway include both infectious and non-infectious stimuli. Toll-like receptors (TLRs), a protein present on the immune cell surface, play a crucial role in this process, by recognizing the pathogen-associated molecular patterns (PAMPs) and damage-associated molecular patterns (DAMPs), this recognition initiates a signaling cascade that activates NF-κβ. Non-infectious stimuli including ROS, cytokines like TNFα and IL-1β, and other stress signals can also cause NF-κβ activation [32, 33].

When NF-κβ stimulates innate immune system cells, such neutrophils and macrophages, which aid in the removal of infections and the start of tissue healing, pro-inflammatory mediators are produced. PAMPs, proinflammatory cytokines generated by these innate immunity cells, and certain TNFSF (tumor necrosis factor receptor superfamily) members bind to specific receptors in the TLR/IL-1R superfamily or the TNFRSF. The IKK complex (Iκβ kinase), which is made up of the three subunits IKKα, IKKβ, and IKKγ (NEMO-NF-κβ essential modulator), is activated by this binding, leading to NF-κβ activation, which drives the innate immune response [34].

NF-κB is also essential for adaptive immune responses; it controls the activation and differentiation of T and B cells. It also plays a role in the development of effector subsets, Th1, Th2, Th17, and regulatory T cells (Tregs), which is supported by co-stimulatory signals from molecules such as CD28. B cells multiply, develop into plasma cells, and release antibodies as a result of NF-κβ activation triggered by antigen detection by the B cell receptor (BCR) [35]. Furthermore, T cell co-stimulatory signals, which are mediated by cytokines like IL-4 and molecules like CD40 ligand, further boost B cell NF-κβ activation, encouraging antibody class flipping and differentiation.

However, persistent or dysregulated activation of NF-κβ can contribute to chronic inflammatory diseases, autoimmune diseases and cancer. In these situations, the negative feedback mechanisms that typically end the inflammatory response may be overpowered by the prolonged NF-κβ activation stimulus [36]. It is this complex signaling cascade that highlights the role of NF-κβ in controlling immunological responses, inflammatory reactions, and the development of cancer (Table 1).

**Table 1:** Therapeutic targeting of inflammation in Prostate cancer

Strategy	Description	Studies & findings	Potential benefits
NSAIDs	Nonsteroidal anti-inflammatory drugs such as Aspirin, Naproxen, Mefenamic Acid, etc	Aspirin: 30% lower risk of PC mortality with higher cumulative use, no association with PC incidence [110, 111]	- Decrease in PC incidence, progression, and recurrence - Apoptosis induction - DNA damage repair - Platelet activity inhibition
		Metformin: Lower PC incidence, improves prognosis but no association with PC incidence [110, 112]	
		Statins: Mixed results on PC risk [110, 113]	
NF-κβ inhibitors	Targeting NF-κβ signaling pathways	Bortezomib: Inhibited cell growth in DU145 PC cell line [114]	- Suppression of inflammatory pathways - Inhibition of tumor growth
		Curcumin: NF-κβ and AP-1 signaling inhibition in PC-3 cells, anti-tumor effect in many cancers including PC [115–117]	
IL-6 Inhibitors	Targeting IL-6 signaling pathways	Siltuximab: Reduced CRPC progression in androgen-dependent PC models. No significant outcome in phase 2 trials [118]	- Potential reduction in castration-resistant progression
STAT3 Inhibitors	Targeting STAT3 signaling pathways	Galiellalactone: Blocked generation of immunosuppressive MDSC-like monocytes, inhibited expression of inflammatory cytokines IL1β, IL10, IL6, reduced expression of indoleamine 2,3-dioxygenase gene, secretion of IL8 and granulocyte macrophage-colony stimulating factor by PC cells [119]	- Reduction of inflammatory cytokines—Inhibition of immunosuppressive monocytes

The promotion of inflammation is mostly dependent on NF-κβ signaling, and chronic inflammation is a major risk factor for PC. Most cancer cells have aberrantly active NF-κβ, which is thought to be responsible for apoptosis suppression, angiogenesis promotion, epithelial-mesenchymal transition/metastasis facilitation, cellular metabolic alteration, anti-tumor immune response suppression, and accelerated cell proliferation.

The abnormal activation of NF-κβ in cancer cells is also linked to the development of malignant tumors in a number of cancer types, including HNSCC (Head and Neck Squamous Cell Carcinoma), breast cancer, colon cancer, and lung cancer [37–40]. Chronic inflammation is a key activator in the metastatic cascade.

Additionally, NF-κβ influences the cells recruited to and forming the tumor microenvironment. NF-κβ becomes activated in particular microenvironment cells upon exposure to PAMPs, endogenous TLR ligands, and unknown ligands through TLR and other receptors. For example, TLR signaling via MyD88-dependent pathways requires TNF receptor-associated factor 6 (TRAF6). When NF-κβ is activated in these cells, proinflammatory cytokines, such as TNF and IL-1, are produced [41]. These cytokines then trigger NF-κβ activation in premalignant or tumor cells, which promotes the synthesis of genes related to angiogenesis, metastasis, proliferation, and survival.

Moreover, EMT, a complex process involving the breakdown of cell–cell adhesion and the gain of mesenchymal traits by epithelial cells, is associated with increased cancer invasion and metastasis. And NF-κβ signaling has been identified as a key player in promoting EMT, in various cancers including PC, contributing to enhanced invasiveness and metastasis.

#### PI3K/AKT pathway

This pathway is important for many cellular processes such as growth, proliferation, survival, motility, metabolism, and immune response regulation. Additionally, inflammation promotes the growth of tumor by stimulating phosphatidylinositol 3-kinase (PI3K) /protein kinase B (PI3K). pathway. For instance, PI3K/Akt signaling triggered by inflammation controls migration and permeability of endothelial cells and affects the course of tumor. By controlling the activity of downstream targets, the PI3K signaling pathway influences the secretion of inflammatory cytokines by cells of the innate immune system.

The primary signaling molecules that activate the PI3K pathway include oncogenes like Ras that attach to the p110 subunit of PI3K, G-protein-coupled receptors (GPCRs), and receptor tyrosine kinases (RTKs). When PI3K is engaged, its catalytic component changes PIP2 into PIP3. When PIP3 is produced, PI3K binds to the pleckstrin homology domain of Akt, resulting in conformational changes that phosphorylate Akt. This phosphorylation process is necessary for the full activation of Akt. Activated Akt moves to the cell membrane from the cytoplasm. NF-κβ and mTOR are two downstream molecular proteins that are either directly or indirectly activated by this cascade, increasing inflammatory responses, cell survival, proliferation, and PC malignancy [42]. The PI3K/AKT pathway plays a role in the development of Multi-Drug Resistance (MDR), partly because of activation of NF-κβ. This pathway may be involved in MDR by inducing PI3K/AKT/NF-κβ activation, which results in cyclin D1 transformation, G1/S phase protein expression, and cell cycle acceleration [42]. Furthermore, uncontrolled PI3K signaling is common in cancer, mainly due to the roles of its catalytic subunits, p110α and p110β. PC is one of several malignancies linked to mutations in the PI3KA gene, which codes for p110α. This gene is essential for endothelial cells because, in addition to controlling proliferation and the cell cycle, it encourages the development of blood vessels that are necessary for tumor growth and metastasis. Ablating p110β significantly reduces Akt activation, leading to a marked decrease in tumor growth in PTEN-deficient prostate cancer models. According to recent research, p110β disruption slows down the onset and development of CRPC, however, p110α inhibition has no effect on PTEN-null CRPC [43].

PTEN is a key regulator of the PI3K/AKT pathway, acting as a tumor suppressor by dephosphorylating PIP3 back to PIP2, which inhibits the pathway. PTEN’s phosphatase activity, which targets both lipids and proteins, helps prevent uncontrolled cell growth and promotes cell death [44]. PTEN mutations disrupt its tumor-suppressive role by either inactivating its phosphatase function or altering its protein-specific activity. Besides regulating PI3K/AKT, PTEN supports genomic stability, cell renewal, senescence, migration, and metastasis within the tumor microenvironment. Glioblastoma, lymphoma, breast, prostate, endometrial, ovarian, colon, and melanoma are among the cancers that have been linked to PTEN mutations [45]. Akt activation phosphorylates a wide range of substrates involved in angiogenesis, metabolism, and cell survival, such as TSC1, TSC2, GSK3, FOXO, p21, p27, caspase-9, BAD, and iNOS. PC and other cancers are characterized by hyperactivation of Akt. PC is caused by hyperactivation of the PI3K/AKT/mTOR system, which is brought on by overexpression of AKT or loss of PTEN in prostate cells, according to research using transgenic and mutant animal models. AKT1, TSC1, and TSC2 (Tuberous Sclerosis Complex 1 and 2), which encode the proteins hamartin and tuberin, respectively, are crucial in PTEN-mediated cancer progression [46]. In particular, it has been demonstrated that PTEN deletion, in conjunction with loss of mTOR or RICTOR (a component of the mTORC2 complex), slows the course of PC [47]. Up to 42% of primary and all metastatic PC samples have aberrant gene expression and changes in PI3K pathway components, based on genomic and transcriptome analyses [48]. PTEN loss and subsequent pathway activation are observed in 40% of primary tumors and 70% of metastatic cases, highlighting the significant role of the PI3K/AKT/mTOR pathway in PC.

#### JAK/STAT signaling pathway

In PC, abnormal and prolonged Janus kinase/signal transducer and activator of transcription (JAK/STAT) activation is associated with tumor development, progression and lineage plasticity. The JAK/STAT signaling system may be activated by a variety of cytokines, growth factors, and hormones, including growth hormone, interleukins, and interferons. This can result in phenotypic changes in a range of tissues and cell types. JAK is transphosphorylated when a ligand attaches to its receptor. Tyrosine residues on the receptor are subsequently phosphorylated by the active JAK, forming a docking site for STAT proteins. Then, through SH2-domain–phospho-tyrosine interactions, JAK phosphorylates STATs at this location, causing them to separate from the receptor and form homodimers or heterodimers. After that, these dimers go to the nucleus, where they attach to the promoters of target genes and control transcription.

Among other ligands, IL-6 stands out as a potent inflammatory cytokine and a crucial regulator of PC progression via JAK/STAT pathway. Elevated serum IL-6 levels have been observed in metastatic PC and CRPC cases compared to healthy men or those with localized disease [49]. A recent study found that, in mice model, a high fat diet elevated IL-6 secretion by prostate macrophages, which triggered STAT3-driven growth of myeloid-derived suppressor cells and fostered a tumor-promoting [50]. Apart from IL-6, there are other drivers that involve the same pathway for induction of PC via inflammation, which includes IL-11, IL-8, Oncostatin M (OSM), and Leukemia inhibitory factor (LIF). OSM and LIF are primarily found in the stromal compartment of the prostate; however, their expression is found to be elevated in the prostate epithelial cells in PC patients [51]. OSM is responsible for EMT, induction of morphological changes, and migration of PC cells through the JAK/STAT3 pathway [52], while ADT- induced LIF, activates STAT3 signaling to promote neuroendocrine differentiation (NED) and CRPC [53]. Similarly, IL-8 plays role in preventing apoptosis in PC cells through the STAT3 pathway [54]. And IL-11 activates STAT3 by binding to its receptor (IL-11Rα), which promotes the proliferation of PC cells leading to oncogenesis [55]. Additionally, studies indicate that JAK/STAT signaling is vital for driving lineage plasticity, a factor that contributes to resistance against androgen receptor (AR)-targeted therapies in PC [56].

### Oxidative stress and reactive oxygen species (ROS)

ROS are chemicals produced by immune cells during a normal inflammatory response to kill pathogens. Elevated ROS levels in cells can damage DNA, increasing the risk of mutations that could lead to cancer. NADPH oxidases (Nox) are crucial for both generating ROS and supporting the growth and maintenance of malignant cells, and significant ROS production takes place in PC [57, 58]. ROS are continuously produced by the body due to immunological reactions, mitochondrial bioenergetics, and oxidative metabolism. Physiological activation of AR has been shown to promote the formation of ROS [59]. This implies that androgen activation in PC cells may contribute to mt-DNA mutations and aging-related processes by increasing ROS formation, which can damage DNA and perhaps influence the mutation rate in cells, including PC cells.

The glutathione peroxidase system eliminates peroxides through the glutathione redox system. This serves as a crucial antioxidant defense mechanism. And glutathione acts as a marker for redox status in various diseases, aging processes, and cell death. It utilizes glutathione peroxidase to catalyze the glutathione redox system to neutralize peroxide [60]. PC mainly inactivates glutathione peroxidase 3 (GPX3), an enzyme that is dependent on selenium and essential for the detoxification of reactive oxidative species. Homozygous and hemizygous deletions of the GPX3 gene were shown to be common in PC samples in 39% of the examined samples. Additionally, Yu et al. found that 90% of the GP X3 exon 1 region is methylated in PC samples [61]. The progression from inflammation to preneoplastic lesions, such as high-grade prostatic intraepithelial neoplasia (PIN), and eventually to prostate cancer (PC) may be influenced by the methylation of the GSTP1 gene. This methylation leads to a loss of the gene's protective function [62].

### Pro-inflammatory tumor microenvironment

**Fig. 3:**
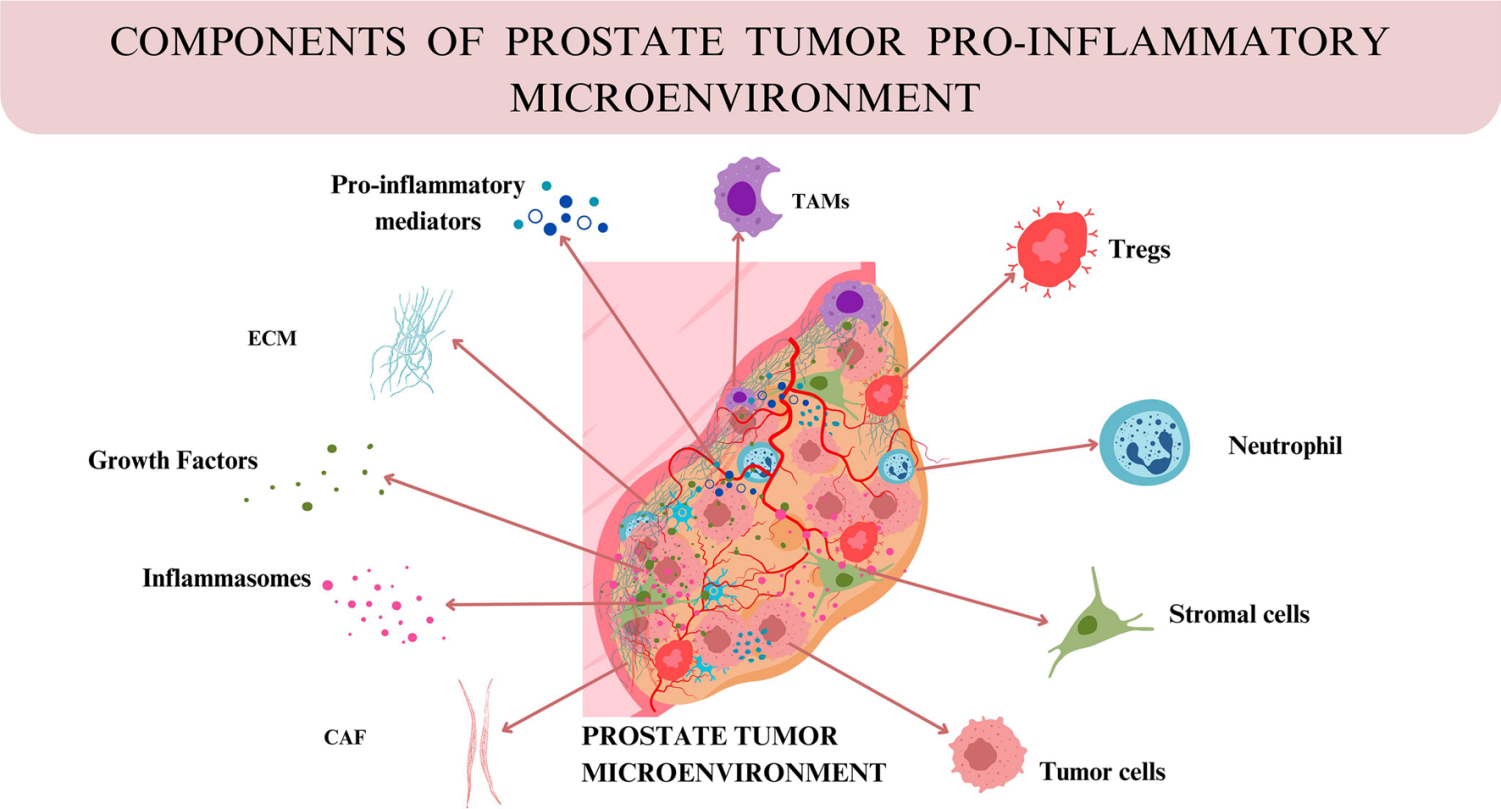
The pro-inflammatory tumor microenvironment of PC, a complex ecosystem of tumor cells, stromal cells, immune cells, cytokines, chemokines, pro-inflammatory mediators, inflammasomes, and a dense extracellular matrix, which collectively drive tumor progression, metastasis and immune evasion

The tumor microenvironment (TME) of PC is a complex ecosystem that includes tumor cells, stromal cells, immune cells, and a dense ECM. This intricate network of cellular and non-cellular components supports tumor development and progression while also contributing to drug resistance. The prostate TME is characterized by a pro-inflammatory environment that influences cancer cell behavior and surrounding stroma. Inflammation results from factors such as chemokines, cytokines, inflammasomes, stromal cells, and fibroblasts secreted by tumor cells and immune cells. This inflammatory setting promotes pro-tumorigenic processes like angiogenesis, invasion, and metastasis (Fig. 3).

#### Growth factors

Growth factors stimulate signaling pathways that allow cancer cells to survive and adapt in the TME. Important growth factors in PC include IL-6, transforming growth factors (TGF-α and TGF-β), epidermal growth factor (EGF), and insulin-like growth factors I and II (IGF-I and IGF-II). Among these growth factors, the overexpression of epidermal growth factor receptor (EGFR) is particularly significant, as it is linked to lower survival rates in PC patients. As PC advances, cells shift from producing EGF to TGF-α, promoting autocrine growth and uncontrolled proliferation [63]. High levels of circulating IGF-I are associated with an increased risk of PC [64]. IL-6 also affects growth, differentiation, and apoptosis in PC by activating the STAT and MAPK signaling pathways. Patients with metastatic PC typically have higher serum levels of IL-6, which may speed up tumor growth over time.

#### Proinflammatory mediators

PGE2 is the most abundant proinflammatory mediator in prostate tissue, with elevated levels in PC [65]. PGE2 is produced by the COX pathway from arachidonic acid and stimulates the growth, multiplication, and metastasis of cancerous cells in addition to upregulating antiapoptotic proteins and immune system regulation. PGE2 stimulates PC cell proliferation by binding to E-prostanoid receptors (EP1, EP2, EP3, and EP4) and modifies kinase pathways such as PI3K/Akt and PKA. CRPC is linked to overexpression of EP4, and EP4 antagonists such as ONO-AE3-208 prevent this progression by controlling androgen receptor activation [66]. NSAIDs that target COX-2 are effective but are limited by cardiovascular side effects [67]. As a downstream player in the PGE2 and COX-2 pathway, EP4 emerges as a promising new target for prostate cancer treatment. Studies show that TLR4 and COX-2 levels are elevated in prostate cancer, and reducing their expression can inhibit cell proliferation, migration, and invasion by lowering p65 phosphorylation. Through the activation of NF-κβ and MAP kinase pathways, TLR4 influences COX-2 regulation in prostate epithelial cells, impacting cancer progression [68].

#### Inflammasomes

Inflammasomes are key regulators of the innate immune response and play an important role in shaping the TME. They regulate inflammation and affect how different cell types behave, influencing tumor growth and immune evasion. Among inflammasome NLRP3 inflammasome is the most extensively studied across cancers [69]. It is a multiprotein complex composed of NLRP3 sensor, ASC (apoptosis-associated speck-like protein) and pro-caspase-1. The activation of the NLRP3 inflammasome is tightly regulated by an upstream signaling cascade [70]. In the initial priming phage of NLRP3 activation TLR plays a crucial role. TLRs are pattern recognition receptors located on the cell surface or within endosomes which recognize PAMPs and DAMPs. Upon recognition of inflammatory triggers TLRs initiate downstream signaling through adaptor proteins such as MyD88 and TRIF, leading to the activation of NF-κB [71]. NF-κB activation in the NLRP3 inflammasome signaling cascade induces the transcription of pro-inflammatory cytokines, including pro-IL-1β and pro-IL-18, thus priming the inflammasome for activation. This priming step is crucial for the subsequent activation of NLRP3. Following NLRP3 activation, caspase-1 is cleaved, leading to the release of mature IL-1β and IL-18, which promote inflammation [71]. The NLRP3 and NLRP12 inflammasomes are activated by various stimuli and help express IL-1 and IL-18 in PC [34]. Dysregulation of inflammasomes, especially NLRP3 and NLRP12, are associated with PC. NLRP12 levels are notably higher in PC tissues than in nearby benign tissues. Moreover, it has been observed that NLRP12 overexpression activates caspase-1 and NF-κβ, which causes macrophages to secrete IL-1β. NF-κβ and IL-1β have both been linked to PC metastasis to the bones [72]. Further a study reported that NLRP3 inflammasome activation promotes the malignant progression of prostate cancer by enhancing cell proliferation, migration, and inhibiting apoptosis through caspase-1 activation [73].

#### Chemokines

Chemokines are chemotactic cytokines that bind to their specific receptors to increase their actions. They are a class of chemo-attractive regulatory proteins that move and arrange leukocytes, hence being essential in coordinating immune responses and inflammation [74]. The chemokine network is intricate, with various chemokines interacting with their receptors to carefully coordinate these immune functions [75]. Cytokines and chemokines influence tumorigenesis directly by regulating tumor cell proliferation, invasion, and metastasis, and indirectly by modulating immune and stromal cells, promoting metastatic niches, and stimulating angiogenesis in the cancer microenvironment [76]. Some important cytokines/chemokines associated with PC include VEGF, IL-6, IL-1, IL-7, CCL2, CXCL12, CXCL16, TGFβ, CXCL1, CXCL8, CX3CL1 and RANKL [77]. Prostate tumorigenesis has been linked to IL-6, a pleiotropic proinflammatory cytokine whose actions are mediated by both autocrine and paracrine processes. It has been discovered to be involved in angiogenesis, EMT, and bone remodeling. Its actions are initiated by binding to the IL-6R receptor and subsequently triggering several signaling pathways, such as the Ras/MAPK, PI3K and JAK/STAT pathways [78]. IL-6 has been identified in multiple studies as a prognostic factor in PC, with patients with metastatic stage having higher serum levels [79]. Chemoattractant CCL2 contributes to metastasis in different types of cancers. It is expressed by endothelial cells, stromal cells, osteoblasts in the bone marrow, and tumor cells [80]. PC cells function both autocrinally and paracrine manner, and they express CCL2 at higher levels. In a study, it was found that chemokine is primarily expressed in patients' primary tumors from PC, and that the production of this chemokine eventually leads to the breakdown of the immune system's tolerance to CCL2 [81]. Members of the CXC family of chemokines, CXCL12 binds to CXCR4 and CXCR7 [82]. PC is associated with elevated expression of CXCL12 and CXCR4, with high CXCR4 expression serving as a marker for bone metastasis [83]. Taichman and associates showed that CXCR4 expression was positive in PC cell lines that had metastasized from the bone [84]. In metastatic prostate cancer, circulating tumor cells (CTCs) express a mix of markers—mesenchymal markers (vimentin, N-cadherin, O-cadherin), stem cell markers (CD133), and epithelial markers (E-cadherin, EpCAM, cytokeratin) [85]. Cytokines like IL-6, IL-7, IL-8, and TGF-β drive EMT, an essential process in cancer spread. Additionally, IL-10 promotes EP4 protein, which helps suppress inflammation triggered by macrophages [86]. In prostate cancer cells, PGE2 activation of EP4 raises the levels of proteins that encourage metastasis [87].

#### Immune cells

The prostate TME shows alterations in the abundance of various immune cell classes. These comprise neutrophils, T cells, and tumor-associated macrophages (TAMs). Between 30 and 50 percent of the immune cells infiltrating the tumor are macrophages [88, 89]. The primary signal transduction pathway that macrophages activate in response to diverse external stimuli is Akt signaling. Both M1 and M2 macrophages have activated Akt signaling; however, the majority of available data indicates that Akt signaling aggravates the M2 condition. Additionally, PI3K signaling also plays a crucial role in T and B cell fate [88]. This pathway, along with other immune-regulating mechanisms, influence TAM behavior. Numerous studies on PC have revealed that TAM infiltration into the TME promotes PC cell migration and proliferation, is linked to the spread of the disease and metastasis and is frequently associated with a low prognosis for patients with metastatic PC [89]. T-cells are major class of tumor-infiltrating lymphocytes in PC [90]. The immunosuppressive cytokines released by Tregs include indoleamine 2, 3-dioxygenase, IL-10, TGF-β, IL-35, CD39, and CD73 [91]. An investigation of Tregs in PC patients revealed that patients' levels of Tregs were considerably greater when PC metastasized to bone than in patients with localized PC [92]. Increased Treg levels in PC patients have been linked to worse survival outcomes, as the immunosuppressive properties of Tregs facilitate tumor progression [93]. Furthermore, it has been demonstrated that neutrophils are significant in the PC microenvironment. When neutrophils are drawn to a site of damage, they release ROS, neutrophil extracellular traps, and proteases such neutrophil elastase. These proteases exacerbate the injury and play a role in the emergence of persistent inflammation. Normally, neutrophils can switch to suppressing the immune system, which in turn regulates the generation of mediators that promote inflammation. Thus, neutrophils are essential in the connection between inflammation and cancer. In a study by Bahig et al. it is reported that increased neutrophil counts are associated with worse overall survival in localized PC [94]. PC stimulates the recruitment of neutrophils. These neutrophils heavily infiltrate prostate tumor regions in bone metastatic PC patients [95].

#### Stromal cells and fibroblasts

Cancer-associated fibroblasts (CAFs) are essential components of the tumor microenvironment and contribute to the tumors’ immunosuppressive nature. They promote PC progression by interacting with M2 macrophages. CAFs induce inflammation and angiogenesis in the TME by releasing cytokines like MCP-1, SDF-1, and CXCL14, which stimulate macrophage infiltration. M2 macrophages stimulate the development of CAFs by inducing EMT in PC cells and triggering new blood vessel formation [96].

Moreover, hypoxia induced by ADT can trigger autocrine TGF-β signaling to be activated. This signaling results in trans-differentiation of CAFs into myofibroblasts that produce CXCL13 [97]. These myofibroblasts then recruit IgA + plasmacytes, which inhibit the activity of cytotoxic T lymphocytes (CTLs) [98]. The interaction of stromal cells, immune cells and tumor cells forms a complex network that contributes to resistance against immune-targeting treatments in PC.

#### Extracellular matrix (ECM)

The ECM is made up of a complex web of proteoglycans, collagen, fibronectin, and laminin. This provides structural and metabolic support to the neighboring cells. It also has a significant impact on the regulation of cell survival, proliferation, and communication. Changes to the ECM can lead to apoptosis in healthy cells, disrupting tissue homeostasis, and promoting tumerogenesis. Inflammation constitutes a pivotal factor in shaping the tumor microenvironment through the induction of angiogenesis and the EMT, leading to ECM remodeling. Inflammatory cells and stromal fibroblasts secrete cytokines, chemokines, and proteases like matrix metalloproteinases (MMPs) that degrade ECM components. This degradation facilitates tumor cell invasion, alters mechanical properties of the ECM, and exposes cryptic ECM-binding sites that further promote metastasis [99]. For example, MMP-2 and MMP-9 are commonly overexpressed in prostate cancer PC and their expression is associated with increased PC aggressiveness and metastasis [100]. The responses also lead to the persistent inflammatory response in the tumor microenvironment, which results in numerous secreted mediators. Inflammatory cells produce a wide range of cytokines, including TNF, IL-2, IL-7, RANTES, and macrophage inflammatory protein-1b. Additionally, growth factors like TGF-β and basic fibroblast growth factor (bFGF) are activated by the chronic inflammatory cascade. Reactive stroma is created when these soluble inflammatory mediators are released into the ECM, activating the stromal cells in the vicinity [101].

The ECM also plays a crucial role in regulating immune cell behavior within TME. Abnormal ECM composition or increased ECM stiffness can create physical barriers limiting infiltration and activity of immune such as cytotoxic T lymphocytes (CTLs) and NK cells. For example, the stiffened ECM in cancerous tissues prevents immune cell migration and function, leading to impairment of the immune response. In addition, ECM components like hyaluronan, tenascin-C and fibronectin bind to integrins and other receptors on immune cells, promoting an immunosuppressive environment that supports tumor progression [102]. For instance, integrin αvβ3 is upregulated in prostate cancer and promotes cell migration via activation of the PI3K/AKT pathway [103].

The ECM fragments released from damaged tissues, especially during inflammation, can recruit TAM while suppressing effector T cells’ actions. This interaction not only impairs immune-mediated tumor clearance but also contributes to the development of resistance to immunotherapies in PC [104]. This chronic inflammation subsequently promotes the development of reactive stroma, a fibrotic ECM structure enriched with activated CAFs and an overproduction of ECM proteins. This reactive stroma substantially modifies the mechanical and biochemical properties of the TME, creating a favorable condition that supports tumor growth and immune evasion.

CAFs, in particular, are vital contributors to ECM remodeling, secreting collagen I and fibronectin that increase ECM stiffness and activate tumor-promoting signaling pathways, including the YAP/TAZ pathway. Such ECM stiffness not only enables survival and motility in cancer cells, but also promotes resistance to standard treatments such as chemotherapy and radiotherapy [100]. Moreover, LPS-mediated TGF-β signaling in the reactive stroma triggers fibroblast-to-myofibroblast trans-differentiation during inflammation. These myofibroblasts actively produce ECM components and chemokines like CXCL13, which attract immunosuppressive cells, including regulatory T cells (Tregs) and IgA + plasmacytes. This creates a reinforcing feedback loop that not only sustains tumor progression but also further facilitates immune evasion and the development of resistance to both chemotherapy and immunotherapy [105]. The disruption of ECM homeostasis in the inflamed TME further enables PC cells to evade anoikis, a form of programmed cell death typically triggered by detachment from the ECM. As a result, these cells become more invasive and migratory, fueling aggressive tumor growth and metastasis. This highlights the ECM’s dynamic role and its critical impact on cancer progression.

### Molecular players that can link inflammation to genetic variations

Gene variants involved in immune response and inflammation pathways might increase the risk of developing PC. For example, SNPs in IL1R2, IL8RB, and TLR4 genes have been linked to a higher risk. Moreover, inheritance of IL1R2 rs11886877 AA variant and the TLR4 rs10759932 variant is associated with a slightly increased PC risk. Moreover, other genetic polymorphisms such as RNASEL, including Glu256X and Met1Ile, have been specifically linked to PC, particularly in specific families [8, 106]. A crucial receptor for innate immunity, TLR4 activates the expression of inflammatory genes to trigger an inflammatory response (e.g., IL1, IL6, and IL8), mostly against Gram-negative bacteria via the signaling pathway of nuclear factor-kB. Cheng and associates. revealed that TLR4 variants were linked to an increased risk of PC [107].

Hereditary mutations are responsible for 5–10% of cases of PC. It has been connected to inherited mutations in multiple genes, such as RNASEL, which was previously known as HPCI; BRCA1 and BRCA2: According to Russo and Giri, there is a threefold greater risk of PC associated with inherited BRCA1 gene mutations. BRCA2 gene mutations are associated with an eight-fold increased risk [108].

## Therapeutic targeting of inflammation in prostate cancer

NSAIDs, like aspirin, naproxen, and mefenamic acid, have been associated with various adverse events, according to multiple studies. However, they are also linked to a lower chance of prostate cancer incidence, development, and recurrence. Research has shown that NSAIDs can inhibit cancer progression by blocking several inflammatory pathways, inducing apoptosis in tumor cells, protecting and repairing damaged DNA, and reducing platelet activity, all of which contribute to their potential protective effects against prostate cancer [109]. A population-based longitudinal cohort study of 388,760 Korean men found that aspirin use for a cumulative duration of 547.5 days or more over 2 years was associated with a 30% reduction in PC mortality; however, aspirin use did not correlate with the incidence of PC. These findings are also consistent with other population-based studies [110, 111]. Furthermore, a decreased incidence of PC was linked to the use of metformin in any amount [110]. He et al. conducted another metanalysis and found no correlation between the use of metformin and the PC incidence, despite the fact that metformin therapy appears to be beneficial in improving the prognosis of PC [112]. Another NSAID statin is also reported to be linked to lower PC risk in some studies, however some studies found no relation of statin and PC risk [110, 113]. More elaborate study to decipher mechanistic implication of these NSAIDs and large scale randomized clinical trials are required to establish the therapeutic effect of these drugs in PC.

Further targeting inflammatory signaling pathways and its components are also being explored as potential therapeutics of PC. According to a 2015 study by Zheng et al., beriberimib treatment significantly slowed down the proliferation of DU145 cell lines of PC in a time and dose dependent manner by inhibiting NF- κβ's ability to transcribe its target genes [114]. The natural component of curry spice turmeric, curcumin, inhibits NF-κβ [115]. Various studies have reported the anti-tumor effect of curcumin against many cancers, including PC [116]. Another study found that curcumin significantly and selectively inhibited the AP-1 signaling as well as NF-κβ signaling pathways in PC-3 cell lines [117]. Additionally, siltuximab, a monoclonal antibody that targets IL-6, has shown promising result in slowing the progression of CRPC in models of androgen-dependent PC. It achieves this by inhibiting IL-6 and its downstream signaling pathways, including phosphorylated STAT3. However, phase 2 clinical trials did not show any significant clinical benefits for patients with metastatic CRPC [118]. Additionally, the STAT3 inhibitor galiellalactone was found to prevent the formation of immunosuppressive myeloid-derived suppressor cell (MDSC)-like monocytes that are induced by prostate cancer cells. It also decreased the levels of inflammatory cytokines, including IL-1β, IL-10, and IL-6. Additionally, galiellalactone lowered the expression of the indoleamine 2,3-dioxygenase gene and reduced the secretion of IL-8 and granulocyte–macrophage colony-stimulating factor by PC cells [119].

In conclusion, targeting inflammation with NSAIDs, specific signaling pathway inhibitors, and monoclonal antibodies shows promise for PC therapy. However, to fully understand their therapeutic potential and refine treatment strategies, more mechanistic studies and large-scale clinical trials are needed.

## Conclusion and future prospects

Chronic inflammation is being increasingly recognized as a key driver in the pathogenesis and progression of PC, with substantial evidence linking it to tumor initiation, development, and metastasis. Substantial evidence links it to tumor initiation, development, and metastasis. This review highlights the roles of inflammatory pathways, such as NF-κB, STAT3, and PI3K/Akt, in shaping the tumor microenvironment, promoting immune evasion, and facilitating EMT. Treatments that target these inflammatory factors show promising results and offer hope for more effective therapeutic strategies. Genetic predispositions, environmental factors, and chronic inflammation interact in complex ways, underscoring the need for a deeper understanding of inflammation’s role in PC. In clinical practice, inflammation remains underrecognized, even though its role in cancer development is well-established. By focusing on inflammatory factors in the tumor microenvironment, researchers can develop more accurate diagnostic methods and targeted treatments. These advancements could help lessen the global burden of prostate cancer and pave the way for better treatment options.

## Supplementary Information

Supplementary material 1.

## Data Availability

No datasets were generated or analysed during the current study.
