# Supplementary material for: The heat of the battle: inflammation’s role in prostate cancer development and inflammation-targeted therapies
Source: Discov Oncol. 2025 Feb 1;16:108. doi: 10.1007/s12672-025-01829-4 (PMC11787145; doi:10.1007/s12672-025-01829-4)
Supplement: Supplementary file 1 — Supplementary material 1. [file 12672_2025_1829_MOESM1_ESM.docx]

**Supplementary Figure Legends**

**Supplementary Figure 1:** Pictorial Abstract: Various inflammatory triggers such as infections, dietary and other factors may contribute to prostatic inflammation, which further triggers prostate cancer development through various molecular pathways, and generation of overall pro-inflammatory tumor microenvironment. Further targeting of these inflammatory mediators could be promising therapeutic approach in prostate cancer management.
